# Persistent Homology Analysis of the Microstructure of Laser-Powder-Bed-Fused Al–12Si Alloy

**DOI:** 10.3390/ma16227228

**Published:** 2023-11-18

**Authors:** Asuka Suzuki, Yusuke Sasa, Makoto Kobashi, Masaki Kato, Masahito Segawa, Yusuke Shimono, Sukeharu Nomoto

**Affiliations:** 1 Department of Materials Process Engineering, Graduate School of Engineering, Nagoya University, 1, Furo-cho, Chikusa-ku, Nagoya 464-8603, Japan; sasa.yusuke.y2@s.mail.nagoya-u.ac.jp (Y.S.); kobashi.makoto@material.nagoya-u.ac.jp (M.K.); 2 Aichi Center for Industry and Science Technology, 1267-1 Akiai, Yakusa-cho, Toyota 470-0356, Japan; masaki_2_katou@pref.aichi.lg.jp; 3 ITOCHU Techno-Solutions Corporation, Toranomon Kamiyacho Trust Tower, Minato-ku, Tokyo 105-6907, Japan; masahito.segawa@ctc-g.co.jp (M.S.); yuusuke.shimono@ctc-g.co.jp (Y.S.); 4 National Institute for Materials Science, 1-2-1, Sengen, Tsukuba 305-0047, Japan; nomoto.sukeharu@nims.go.jp

**Keywords:** additive manufacturing, powder bed fusion, Al–Si alloy, persistent homology, topological data analysis

## Abstract

The laser powder bed fusion (L-PBF) process provides the cellular microstructure (primary α phase surrounded by a eutectic Si network) inside hypo-eutectic Al–Si alloys. The microstructure changes to the particle-dispersed microstructure with heat treatments at around 500 °C. The microstructural change leads to a significant reduction in the tensile strength. However, the microstructural descriptors representing the cellular and particle-dispersed microstructures have not been established, resulting in difficulty in terms of discussion regarding the structure–property relationship. In this study, an attempt was made to analyze the microstructure in L-PBF-built and subsequently heat-treated Al–12Si (mass%) alloys using the persistent homology, which can analyze the spatial distributions and connections of secondary phases. The zero-dimensional persistent homology revealed that the spacing between adjacent Si particles was independent of Si particle size in the as-built alloy, whereas fewer Si particles existed near large Si particles in the heat-treated alloy. Furthermore, the first principal component of a one-dimensional persistent homology diagram would represent the microstructural characteristics from cellular to particle-dispersed morphology. These microstructural descriptors were strongly correlated with the tensile and yield strengths. This study provides a new insight into the microstructural indices describing unique microstructures in L-PBF-built alloys.

## 1. Introduction

The laser powder bed fusion (L-PBF) process has been widely applied or investigated as a representative additive manufacturing technology [1,2,3,4]. The L-PBF process makes it possible to seamlessly manufacture complex geometrical metallic parts [5]. Both the complexity of manufacturable parts and the controllable microstructures are characteristics of the L-PBF process. Laser scanning to the powder bed during the L-PBF process rapidly and locally increases the temperature, melts the metal powder, and forms the semi-ellipsoidal melt pools with a high thermal gradient of over 10^7^ K/m [6]. Subsequently, the laser-scanned regions are rapidly cooled and solidified to form characteristic microstructures including a single crystal-like texture [7,8], metastable phase [9,10,11], and supersaturated solid solutions [12,13].

Al–Si-based alloy series are widely used for the L-PBF process due to their good processability and low cost [11,14,15,16,17,18]. In particular, AlSi10Mg and Al–12Si alloys are the most common Al–Si-based alloys in the L-PBF process [9,10,11,19,20,21,22,23,24,25,26,27,28,29,30,31,32,33,34,35,36,37,38,39,40,41,42,43]. These alloys, processed by the L-PBF process, exhibit a higher tensile strength compared with the alloys manufactured by conventional cast processes [9]. That is why the dominant contributors to the high strength of these alloys have been investigated [9,20,42,43]. The microstructure of these alloys is hierarchical, and the melt pool structure is the most macroscopic [40]. In the melt pool structure, <001>-oriented α crystal grains are elongated to the center of the melt pools [40]. More microscopically, the cellular microstructure in which the elongated primary α phase is surrounded by the eutectic Si networks exists [40]. The primary α phase contains highly concentrated solute Si (2–3 mass%), exceeding the solubility limit of the Al–Si binary system (~1.5 mass%) [9,10,11]. Post-heat treatments significantly change the microstructures. Low-temperature direct aging at around 100 °C introduces nano Si precipitates from the supersaturated solid solution and improves the tensile strength–ductility balance [37,38,39]. Heat treatments over 200 °C slightly affect the α grain size and orientations, whereas the cellular microstructure changes to a Si particle-dispersed microstructure with heat treatments [40,41]. The solute Si content gradually approaches the equilibrium content at each temperature with heat treatments. As a result, the strength is significantly reduced, and ductility is improved by the heat treatments. Recently, in-situ synchrotron X-ray diffraction measurements during tensile testing have been carried out for L-PBF-built AlSi10Mg and Al–12Si alloys [42,43]. The phase stress is highly partitioned into the Si phase in the as-built alloys, which dominantly contributes to the high strength of these alloys. After the heat treatment at 300 °C for 2 h, the phase stress of the Si phase is reduced but still partitioned. After the heat treatment at 530 °C for 6 h, the stress partitioning into the Si phase is less significant [43]. Although the mechanisms for the significant stress partition into the Si phase have not been sufficiently clarified, the morphology and/or spatial distribution of Si particles would be important for the load transfer effect.

The microstructural features are quantitatively analyzed using the fraction, size, number density, and shape of the secondary phases. However, these general microstructural descriptors are not sufficient to quantitatively investigate the structure–property relationship of L-PBF-built Al–Si alloys due to the unique load transfer effect. It is necessary to quantitatively analyze the spatial distribution of the Si phase and the cellular microstructural morphology. Liu et al. quantified the cellular microstructure using the shape and size indices of cells in the L-PBF-built AlSi10Mg alloy [44]. Using these microstructural descriptors, a prediction model with a high accuracy (the square of the determination coefficient over 0.92) was achieved. Although the study demonstrated significant progress in quantifying the cellular microstructure of the L-PBF-built Al–Si alloys, it is difficult to consistently quantify the different microstructural characteristics without cellular morphology. For example, 530 °C/6 h heat-treated AlSi10Mg and Al–12Si alloys exhibited the Si particle-dispersed microstructure [40,41], and the cell shape and size indices are hard to define. The microstructural descriptors that can comprehensively represent the cellular and particle-dispersed microstructures will help with understanding the microstructure–property correlation, not only for Al–Si alloys, but also other alloy systems with cellular microstructures [45].

The data science approaches including a convolutional neural network (CNN) and persistent homology (PH) have been applied to the image analyses [46,47,48,49,50,51,52]. A CNN can be used for segmenting or annotating images [46] and predicting the properties related to the images [47]. In addition, gradient-weighted class activation mapping (Grad-CAM) has been used for explaining the CNN predictions with low interpretability [48]. However, it is difficult to extract the microstructural descriptors that can be intuitively understood. The PH analysis is one of the topological data analysis methods that can analyze spatial distributions and connections between atoms, phases, particles, and so on. PH analysis has revealed the difference in the atomic arrangement between glass and liquid, the arrangement of particles in the particle-packed structures, perlite microstructures, and so on [48,49,50,51,52]. When the two-dimensional micrographs containing secondary-phase particles are analyzed by PH, zero-dimensional (PD0) and one-dimensional (PD1) persistent diagrams are obtained. PD0 and PD1 analyze zero-dimensional and one-dimensional holes, which correspond to the connection and ring structure of particles. The connection of particles (PD0) represents the size and shape of particles and the distance between adjacent particles. The ring structure of particles (PD1) represents the spatial arrangement of three or more particles. It was expected that PH could analyze spatial distributions of Si particles and the characteristics of cellular or particle-dispersed microstructures. In the present study, the microstructure in the L-PBF-built and subsequently heat-treated (300 °C/2 h and 530 °C/6 h) Al–12Si alloys were analyzed using PH. From the persistent homology diagrams, PH microstructural descriptors were quantified. The correlations between PH microstructural descriptors and mechanical properties were also analyzed to clarify the usefulness of the PH descriptors.

## 2. Materials and Methods

A gas-atomized Al–12Si (mass%) alloy powder was used as a raw material. The detailed composition and morphology of the powder were described in our previously reported studies [41,53]. Cubic samples with a size of 15 × 15 × 15 mm^3^ were fabricated using the powder and an L-PBF apparatus (Prox DMP200, 3D systems, Rock Hill, SC, USA). A laser with a power of 191 W, a spot size of approximately 0.1 mm, and a wavelength of 1070 nm was applied at a scan speed of 1.2 m/s, while sliding a scan line at a hatch spacing of 0.1 mm. The powder layer thickness was set at 0.03 mm, and the scan direction was changed by 90° on each layer. The processing conditions were previously optimized so that the dense products with a relative density of over 99.5% were obtained [11].

**Figure 1:**
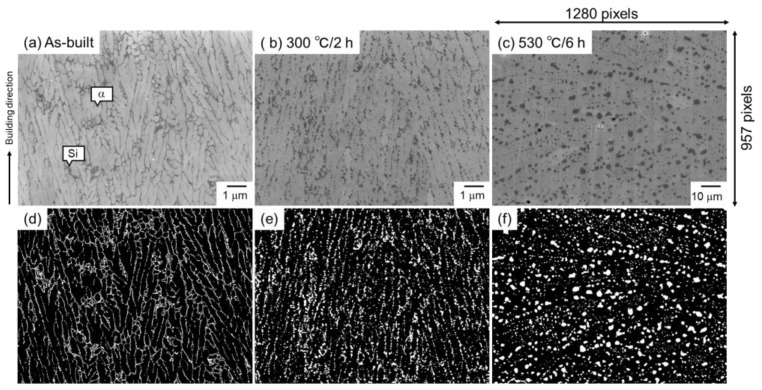
Representative microstructure and corresponding binarized images of (**a**,**d**) as-built and subsequently heat-treated ((**b**,**e**) 300 °C/2 h and (**c**,**f**) 530 °C/6 h) Al–12Si alloys.

The as-built samples were heat-treated at 300 °C for 2 h or 530 °C for 6 h in order to vary the microstructural morphology. The heat treatment conditions were selected based on the previous study [41]. The heat treatment at 530 °C for 6 h was carried out to obtain the particle-dispersion microstructure. The heat treatment at 300 °C for 2 h was adopted to obtain the microstructure between cellular and particle-dispersed structures. The as-built and heat-treated samples were cut into small pieces and ion-polished (Cross-Section Polisher, JEOL, Tokyo, Japan). The microstructural observations were carried out using field emission scanning electron microscopy (FE-SEM, JSM-7401, JEOL, Tokyo, Japan). The image size was fixed at 1280 × 1024 pixels and cropped to 1280 × 957 pixels before image analyses. The representative SEM images are displayed in Figure 1a–c. The as-built Al–12Si alloy exhibited a cellular microstructure in which the elongated α phase was surrounded by a eutectic Si network. When the alloy was heat-treated at 300 °C for 2 h, the eutectic Si phase grew and was granulated. However, the microstructural morphology that was elongated parallel to the building direction was retained after exposure at 300 °C. When the alloy was exposed to 530 °C for 6 h, a significant coarsening of the Si phase occurred (note that the scale bar indicates a 10-times-longer length), and the cellular microstructural morphology of the as-built alloy completely disappeared. The microstructural morphology of the 530 °C/6 h-heat-treated alloy was considered to be particle-dispersion. These microstructural characteristics are very consistent with the previous reports [41]. The microstructural images were binarized using Trainable Weka Segmentation (TWS) [54], plugged into image analysis software (Image J Ver. 1.53t, National Institutes of Health, Bethesda, MD, USA), and are shown in Figure 1d–f. The general microstructural descriptors (area fraction, number density, equivalent diameter, and circularity of Si particles) were analyzed by the image analysis software and are summarized in Table 1. The area fraction of the Si phase in the as-built alloy was consistent with a previous study [11], indicating the reliability of the binarization. In this study, persistent homology analyses were applied to Si phases in the binarized images (white parts in Figure 1d–f) using software (Homcolud3.5.2) [55], carried out in Python 3.8.17. Three images were used for each sample. Note that the image with different magnification (10-times-lower magnification) was used for the 530 °C/6 h-heat-treated alloy because a significantly small number of Si particles were analyzed if the same magnification was applied. When the value with a dimension of length was quantified, pixel size was multiplied to reflect the real scale. In addition, to understand what information can be obtained by the persistent homology analyses of the microstructural image, an artificially constructed imaginary microstructural image (1189 × 825 pixels) was also analyzed.

## 3. Results

**Figure 2:**
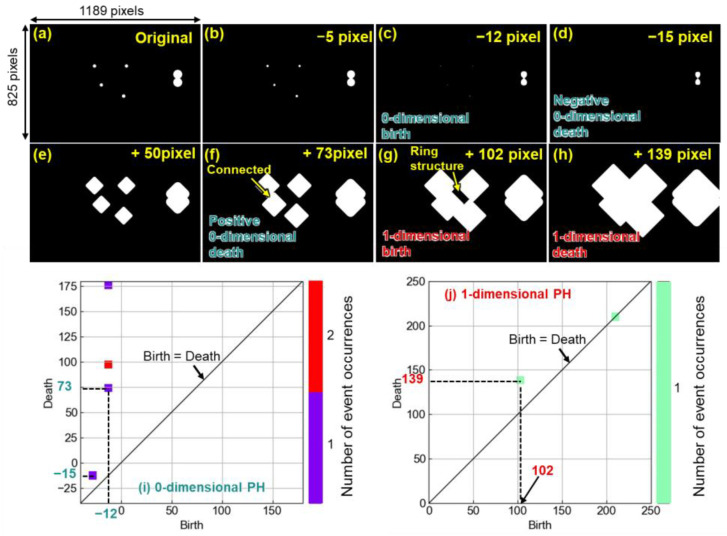
Persistent homology analyses of imaginary microstructural image containing secondary-phase particles: (**a**) Original images and images containing (**b**) −5-, (**c**) −12-, and (**d**) −15-pixel shrunk secondary particles or (**e**) +50-, (**f**) +73-, (**g**) +102-, and (**h**) +139-pixel thickened secondary particles. Corresponding (**i**) zero-dimensional (PD0) and (**j**) one-dimensional (PD1) persistent homology diagrams.

Figure 2 shows the results of persistent homology analyses of the artificially constructed imaginary microstructure. The imaginary microstructure contained four spherical particles and one necked particle (Figure 2a). In the persistent homology analyses, the white particles were shrunk or thickened by one pixel (Figure 2b–h), and the birth or death events were observed. Based on the event observations, zero-dimensional and one-dimensional persistent homology diagrams (PD0 and PD1) (Figure 2i,j) were created. When the particles were thickened, the spherical morphology changed to a square morphology (Figure 2e–h). This is due to thickening based on the Manhattan distance [56]. The Manhattan distance is defined as the minimum number of times a boundary is passed when moving from one position to another; in the PH analysis, black pixels with a Manhattan distance of 1 from the boundary between white and black pixels were changed to white pixels (see Supplementary Materials, Figure S1). The curvature of the original particle was then reduced, and its morphology changed from spherical to square. If the Euclidean distance was employed, the spherical shape could be maintained after thickening. However, the computational cost would be higher, while the information obtained would not change much. Therefore, the Manhattan distance was adopted in this study. In both diagrams, the horizontal and vertical axes correspond to the thickened or shrunk pixels at which birth and death occurred. The diagonal lines in the diagrams indicate that the pixels at birth equal the pixels at death, above which, all the data are always plotted. In PD0, one data point was plotted at the birth of −12 pixels and the death of 73 pixels (Figure 2i). When the spherical particles were shrunk by −12 pixels, the particles almost disappeared (Figure 2c) (if shrunk by −13 pixels, the particles completely disappeared). This means that the spherical particles were born at the stage of −12 pixels, and the information was plotted at the birth of −12 pixels. Thus, the birth value in the PD0 is related to the size of the particles. By contrast, when the particles were thickened by 73 pixels (Figure 2f), the adjacent two particles were connected. This means that the connection of the original particles disappeared, and that is why the information was plotted at the death of 73 pixels. The death value in the PD0 is related to the spacing between adjacent particles. In the PD0, a negative death value also existed, e.g., the death value of −15 pixels. When the necked particles were shrunk by −16 pixels, the necked particles were separated into two particles. In other words, the connection of the isolated particles disappeared at the stage of −15 pixels (Figure 2d). Therefore, the negative death value is related to the morphology of the particles. As the value is negatively low, the particles are more necked because a slight shrinkage causes the particles to become separated. The color of the plots classified the number of event occurrences at the same time. The death of around 100 pixels occurred at two positions at the same time. Two isolated particles at the stage of +73 pixels were connected to each other at +100 pixels; at the same time, the connected particles were further contacted with two particles connected at the stage of +73 pixels. In PD1, one data point was plotted at the birth of 102 pixels and the death of 139 pixels (Figure 2j). When the spherical particles were thickened by 102 pixels, four particles made a ring structure (an isolated black part surrounded by thickened white particles) (Figure 2g). The information showing that the ring structure was born was plotted at the birth of 102 pixels. When the four particles were further thickened, the isolated black part was covered with thickened white particles (Figure 2h). The information demonstrating that the ring structure disappeared was plotted at the death of 139 pixels. Thus, the birth and death values in the PD1 are related to the spatial distributions of particles. It was expected that the persistent homology analyses of the microstructures shown in Figure 1 would provide the size, morphology, spacing, and spatial distributions of Si particles.

**Figure 3:**
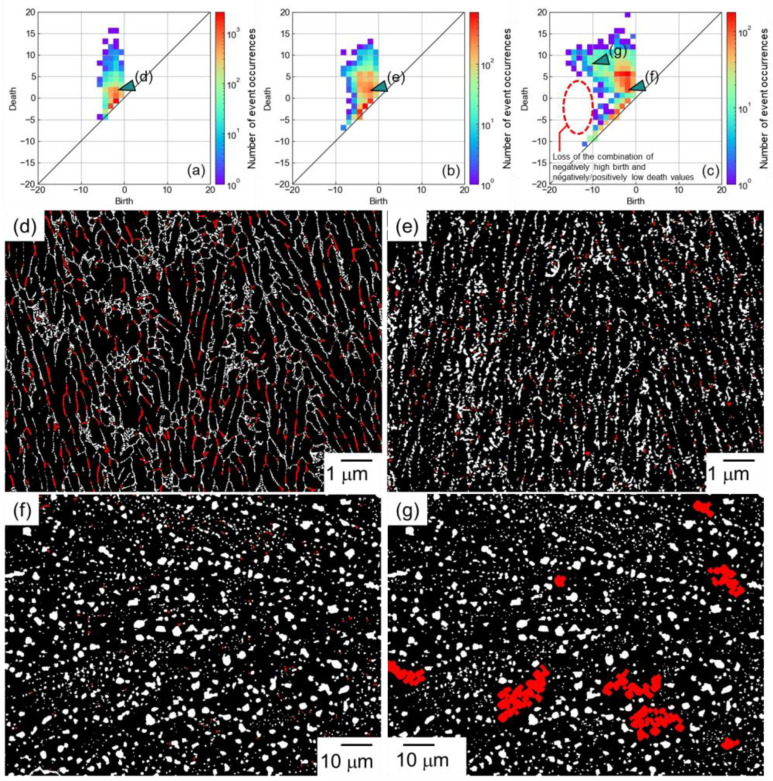
PD0 for (**a**) as-built, (**b**) 300 °C/2 h-heat-treated, and (**c**) 530 °C/6 h-heat-treated Al–12Si alloys. The locations corresponding to (birth, death) = (**d**–**f**) (−2, 2) and (**g**) (−10, 8) are highlighted in the binarized images of (**d**) as-built, (**e**) 300 °C/2 h, and (**f**,**g**) 530 °C/6 h-heat-treated alloys.

Figure 3 shows the PD0 for the (a) as-built, (b) 300 °C/2 h-heat-treated, and (c) 530 °C/6 h-heat-treated Al–12Si alloys. In the PD0 for the as-built alloy, the birth and death values were in the range of −6 to −1 pixels and −5 to 15 pixels, respectively (Figure 3a). In the PD0 for the 300 °C/2 h-heat-treated alloy, the birth value range was widened to −8 pixels, reflecting the growth of the Si particles (Figure 3b). The death value range was in the range of −7 to 13 pixels. To compare the spacing between the Si particles of the as-built and 300 °C/2 h-heat-treated alloy, the locations corresponding to (birth, death) = (−2, 2) (corresponding to small Si particles in a narrow spacing) are highlighted in red in Figure 3d,e. The highlighted parts were frequently observed at the eutectic microstructure of the as-built alloy, whereas the numbers were obviously decreased in the 300 °C/2 h-heat-treated alloy. This indicated that the spatial distributions of Si particles became sparse by the 300 °C/2 h heat treatment. In the PD0 for the 530 °C/6 h-heat-treated alloy, the birth and death values were in the range of −12 to −1 pixel and −13 to 17 pixels, respectively (Figure 3c). Considering that a 10-times-lower magnification image was used, the values corresponded to −120 to −10 pixels and −130 to 170 pixels for the as-built and 300 °C/2 h-heat-treated alloys, respectively, indicating that a significant coarsening (size and spacing increment) occurred with the 530 °C/6 h heat treatment. Interestingly, the combination of a negatively higher birth value and positively/negatively lower death values did not exist (indicated by the red broken circle in Figure 3c), which was different from the as-built and the 300 °C/2 h-heat-treated alloy. In particular, the combination of negatively higher birth and positively lower death values corresponded to large Si particles with a close spacing. The disappearance of the combination indicated that fewer Si particles existed near large Si particles. The locations corresponding to (birth, death) = (−2, 2) (corresponding to small Si particles in a narrow spacing) and (−10, 8) (corresponding to large Si particles in a wide spacing) are highlighted in Figure 3f,g. From this figure, it was confirmed that small particles existed in a narrow spacing, whereas few particles existed near larger particles. This would be caused by the coarsening (Ostwald ripening), meaning that small Si particles would dissolve into the matrix and would be used for increasing the size of large Si particles [57]. The persistent homology could help with the understanding of the spatial distribution of particles during the growth and coarsening stages (generally discussed as only the average size, volume fraction, and number density of particles) although systematic evaluations will be needed.

**Figure 4:**
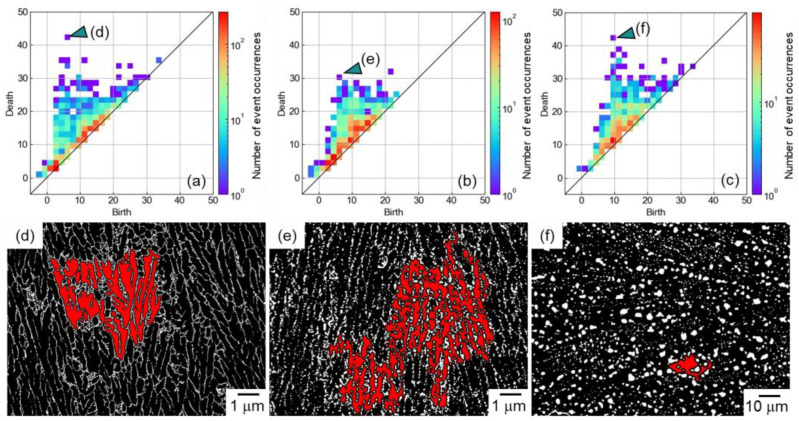
PD1 for (**a**) as-built, (**b**) 300 °C/2 h-heat-treated, and 530 °C/6 h-heat-treated Al–12Si alloys. The locations corresponding to the birth/death combination indicated in (**a**–**c**) are highlighted in the binarized images of (**d**) as-built and (**e**) 300 °C/2 h, or (**f**) 530 °C/6 h-heat-treated Al–12Si alloys.

Figure 4 shows the PD1 for (a) as-built, (b) 300 °C/2 h-heat-treated, and (c) 530 °C/6 h-heat-treated Al–12Si alloys. In the PD1, the combination of a small birth value and a large death value indicates a large ring structure. For example, the plots indicated by arrows in Figure 4a–c have a small birth value and a large death value. The locations corresponding to the plots are highlighted in red in the binarized images shown in Figure 4d–f. In the as-built alloy, the ring structure corresponded to the cellular microstructure composed of the α phase surrounded by the eutectic Si network. This indicated that the PD1 included information regarding the cellular microstructural morphology. In the 300 °C/2 h-heat-treated alloy, the ring structure appeared to comprise the cellular microstructural morphology (elongated α phase surrounded by Si particles). This indicated that the 300 °C/2 h-heat-treated alloy would partially contain the cellular microstructural morphology of the as-built alloy, although the eutectic Si network was separated and granulated. In the 530 °C/6 h-heat-treated alloy, the ring structure did not exhibit the cellular microstructure and was isotropically formed by the dispersed Si particles. This indicated the cellular microstructural morphology would completely disappear and change to the particle-dispersed microstructure.

**Figure 5:**
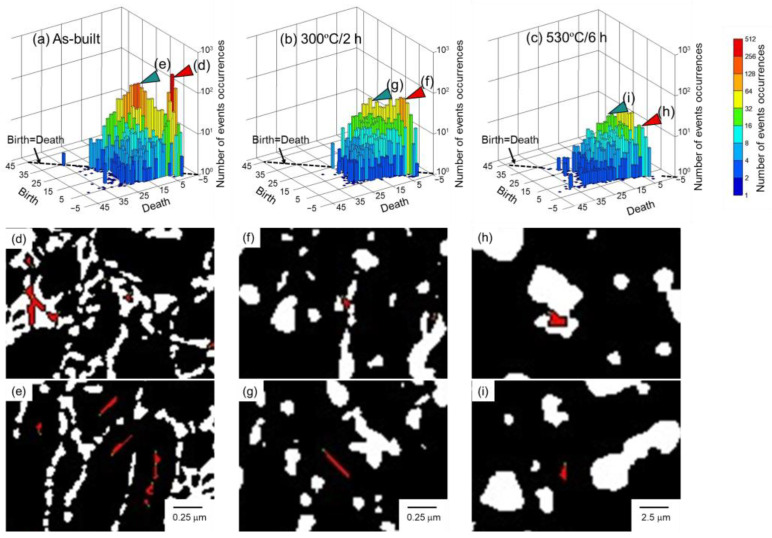
Three-dimensional plots for PD1 of (**a**) as-built, (**b**) 300 °C /2 h-heat-treated, and 530 °C /6 h-heat-treated Al–12Si alloys. Local locations corresponding to the birth/death combination indicated in (**a**–**c**) are highlighted in the binarized images of (**d**–**i**).

To further analyze the PD1, three-dimensional plots of PD1 that have the number of event occurrences as the z-axis were created and are shown in Figure 5. In the PD1 of the as-built alloy, numerous event occurrences were observed near the diagonal broken line (Figure 5a), which indicates birth = death. This indicated that the ring structures disappeared by thickening the Si particle by one pixel from birth. As an example, the locations corresponding to (birth, death) = (2, 3) (indicated by a red arrow) and (12, 13) (indicated by a green arrow) are locally highlighted in red and shown in Figure 5d–i. The combinations of (birth, death) = (2, 3) were frequently observed at the eutectic Si network (Figure 5d). The fine eutectic morphology resulted in small ring structures. The combination of (birth, death) = (12, 13) was observed inside the α phase (Figure 5e). The small ring structure was formed by a relatively necked α phase. At such a part, the thickened Si particles isolated the small α phase but deleted the ring structure by further thickening one pixel. Both the parts were characteristics of the cellular microstructure of the as-built alloy. After the 300 °C/2 h heat treatment, the eutectic Si network was separated and granulated, resulting in a significant reduction in the ring structure formed by the eutectic microstructure, although neighboring Si particles (presumably a trace of the eutectic structure) occasionally formed the ring structure (Figure 5f). In addition, the reduction in the number density of Si particles at the eutectic structure reduced the small ring structure formed inside the α phase (Figure 5g). The 530 °C/6 h heat treatment made the eutectic microstructure and corresponding ring structure disappear. The small ring structure was slightly observed with the irregular-shaped Si particles (Figure 5h). The small ring structure inside the α phase was also reduced along the disappearance of the cellular microstructure (Figure 5i). As a result, the heat treatments reduced the number of event occurrences near the diagonal line of birth = death and leveled the distribution of the birth/death combinations (Figure 5b,c).

## 4. Discussion

**Figure 6:**
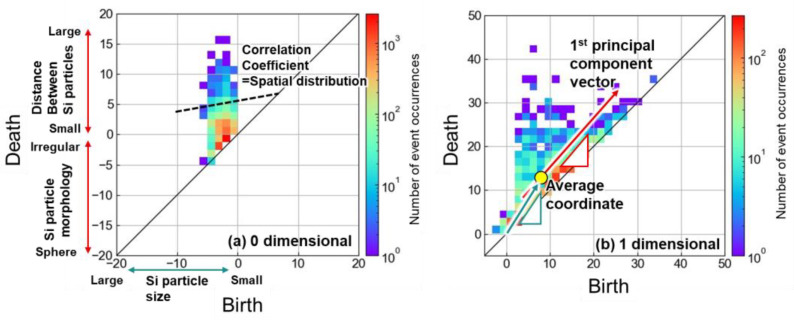
Methodologies for obtaining representative values for describing microstructure from (**a**) PD0 and (**b**) PD1.

The PD0 and PD1 were useful for interpreting the microstructural characteristics. However, to investigate the structure–property relationship, it is necessary to extract some representative values from the PD0 and PD1. Figure 6 illustrates how the representative values were quantified in this study. In the PD0, the birth value corresponded to the size of the Si particles (Figure 6a); therefore, the average birth value was calculated ($\bar{B0}$). The average positive death value was also calculated to evaluate the distance between adjacent Si particles ($\bar{D0+}$). The negative death value contains information on the morphology of Si particles. As the death value was negatively high, the morphology of the Si particle was closer to the sphere. However, the negative death value itself was affected by the size of Si particles. When two Si particles have the same necked shape but different sizes, the larger particle has a negatively higher death value. Therefore, the ratio of the negative death value to the birth value (corresponding to the size) was averaged as the shape descriptor of the Si particles ($\bar{D0-/B0}$). Finally, the correlation coefficient of the birth value and positive death value (*ρ*_B0, D0+_) were quantified. As is shown in Figure 3c, the heat treatment at 530 °C for 6 h generated the spatial distributions of Si particles and fewer particles existed near large particles. It was expected that the correlation coefficient would catch the tendency. From the PD1, it was expected that the microstructural morphology would be quantified. In the PD1, the balance between the birth and death values was important because the information contained varied depending on how far away from the diagonal line the plot was (Figure 4 and Figure 5). First, the average birth and death values were calculated ($\bar{B1}$ and $\bar{D1}$), and their ratio was used as a descriptor ($\bar{D1}/\bar{B1}$). The ratio indicated the slope of the line connecting the origin with the representative coordinates of PD1, representing how far away from the diagonal line the representative coordinate was (Figure 6b). In addition, the principal component analysis was carried out on the PD1. The principal component analysis finds principal components with the directions of variation in data being maximized and orthogonal to each other. The persistent homology diagram is a two-dimensional plot; therefore, two principal components (PC1 and PC2) were determined. The slope of PC1 was quantified (*a*_PC1_), representing how far away from the diagonal line the maximum variation direction was.

**Figure 7:**
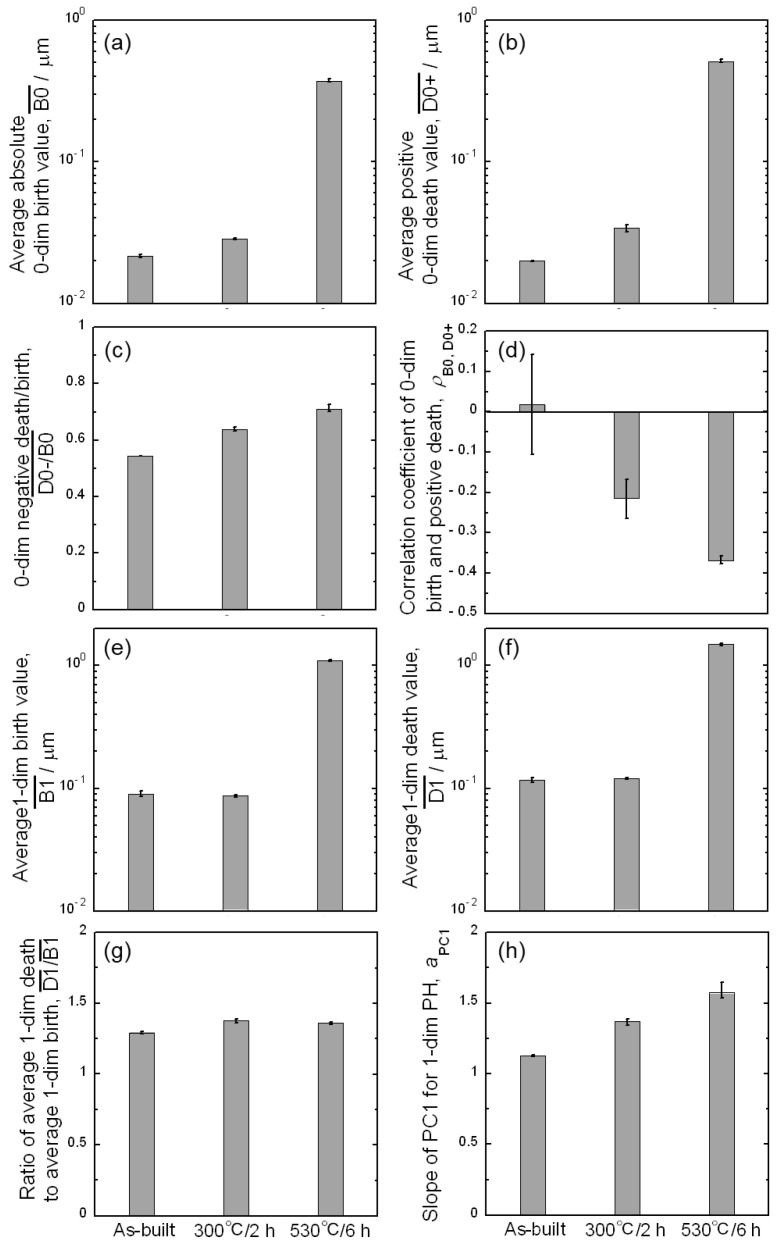
Representative microstructural descriptors obtained from PD0 and PD1: (**a**) Average absolute zero-dimensional birth value, (**b**) average positive zero-dimensional death value, (**c**) average zero-dimensional death/birth ratio with negative death value, (**d**) correlation coefficient between zero-dimensional birth and positive death values, average one-dimensional (**e**) birth and (**f**) death values, (**g**) slope of average one-dimensional death/birth vector, and (**h**) slope of the first principle component (PC1) for one-dimensional persistent homology diagram.

The representative values extracted from the persistent homology diagrams are summarized in Figure 7. In Figure 7a,b,e,f, birth or death values have been multiplied by the pixel size to give the dimensions in μm. The error bar indicates the maximum and minimum values analyzed using three images for each sample. The variation between maximum and minimum values was sufficiently small compared with the change in each descriptor with heat treatments, indicating good reproducibility. The average zero-dimensional birth and positive death ($\bar{B0}$ and $\bar{D0+}$) slightly increased with the 300 °C/2 h heat treatment and significantly increased with the 530 °C/6 h heat treatment (Figure 7a,b). These corresponded to the growth and coarsening of the Si particles. The average negative death/birth ratio ($\bar{D0-/B0}$) increased monotonically by the heat treatments (Figure 7c), indicating that the irregular-shaped Si particles at the eutectic structure changed to a more spherical morphology. The correlation coefficient between the birth and positive death value (*ρ*_B0, D0+_) of the as-built alloy was almost zero (Figure 7d), indicating that the spatial distribution of Si particles was not related to the particle size. The error bar of the data was relatively large because the small correlation makes the correlation coefficient more likely to vary. After the heat treatments, *ρ*_B0, D0+_ exhibited negative values and smaller error bars. Fewer Si particles tended to exist near large Si particles, especially after exposure to 530 °C for 6 h.

The average birth and death values in PD1 ($\bar{B1}$ and $\bar{D1}$) did not change under the heat treatment at 300 °C for 2 h (Figure 7e,f). The heat treatment at 530 °C for 6 h provided average values that were one order higher. The ratio of the average death to average birth values ($\bar{D1}/\bar{B1}$) was almost constant and independent of the heat treatment (Figure 7g). The ratio of the average death to average birth values did not represent microstructural characteristics and could be a common value in various microstructures. By contrast, the slope of the PC1 (*a*_PC1_) increased monotonically by applying the heat treatments (Figure 7h). The *a*_PC1_ for the as-built alloy was approximately 1.1 and increased to approximately 1.5 after heat treatment at 530 °C for 6 h. The *a*_PC1_ would represent the microstructural morphology. In the as-built alloy, the death and birth combinations were highly distributed along the diagonal line of birth = death (Figure 5a). The distribution was caused by the formation of ring structures at the eutectic structure and inside the primary α phase. The distribution provided the variation in data points along the diagonal line, resulting in an *a*_PC1_ close to 1. The distribution was leveled by the heat treatments (Figure 5b,c), resulting in an increment in the *a*_PC1_ to 1.5. The *a*_PC1_ could take values from 1.1 to 1.5 by changing the microstructural characteristics from a cellular to particle-dispersed morphology. Interestingly, the 300 °C/2 h-heat-treated alloy exhibited an intermediate value (approximately 1.3) between the as-built and the 530 °C/6 h-heat-treated alloys. This indicates that the alloy would quantitatively contain the characteristics of both the cellular and particle-dispersed morphologies, which seems to be in accordance with the qualitative microstructural images. Thus, it is not only the size, spacing, shape, and spatial distributions of Si particles that are quantified using the persistent homology analyses, but also the microstructural morphology (cellular or particle-dispersion).

**Figure 8:**
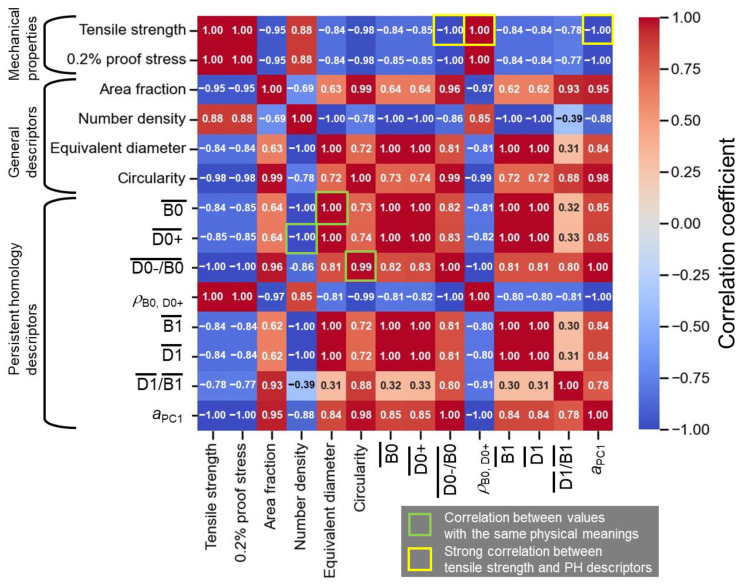
Heatmap showing the correlation between mechanical properties, general microstructural descriptors, and persistent homology descriptors.

It is important to investigate the correlation between the microstructural descriptors and mechanical properties. Figure 8 presents the correlation table of the mechanical properties, general microstructural descriptors (area fraction, number density, equivalent diameter, and circularity of Si particles), and persistent homological descriptors (Figure 7). The tensile strength (463 MPa for the as-built alloy, 299 MPa for the 300 °C/2 h-heat-treated alloy, and 164 MPa for the 530 °C/6 h-heat-treated alloy) and 0.2% proof stress (313 MPa for the as-built alloy, 199 MPa for the 300 °C/2 h-heat-treated alloy, and 104 MPa for the 530 °C/6 h-heat-treated alloy) reported in the previous literature [41] were used here. The $\bar{B0}$, $\bar{D0+}$, and $\bar{D0-/B0}$ values strongly correlated with the equivalent diameter, the number density, and the circularity (highlighted by green rectangles), respectively, which have the same physical meanings. Since only the three types of samples (as-built, 300 °C/2 h-heat-treated, and 530 °C/6 h-heat-treated alloys) were analyzed, almost all of the microstructural descriptors correlated well with the mechanical properties. Although more samples need to be analyzed, the D0−/B0, *ρ*_B0, D0+_, and *a*_PC1_ exhibited an especially strong correlation, reaching −1 or 1 (highlighted by yellow rectangles). These descriptors indicated the shape and spatial distributions of Si particles and the microstructural morphology. Recent in-situ synchrotron X-ray diffraction analyses revealed that stress was partitioned into Si phases in the as-built alloy [42,43], leading to its high strength. The stress partitioning was less pronounced by applying the heat treatments. Although the load transfer mechanisms have not been sufficiently clarified, the distributions of Si particles and/or microstructural morphology would contribute to highly partitioned stress. Therefore, the strong correlation of *ρ*_B0_, _D0+_, and *a*_PC1_ with the mechanical properties could be reasonable. These microstructural descriptors would be useful for investigating the microstructure–mechanical property relationship of the alloys with cellular and particle-dispersed microstructural morphology in greater depth.

## 5. Conclusions

In the present study, the microstructures of laser-powder-bed-fused and subsequently heat-treated (300 °C/2 h or 530 °C/6 h) Al–12Si alloys were analyzed by persistent homology analysis (a topological data analysis method). The microstructural characteristics of the as-built and 530 °C/6 h-heat-treated alloys were qualitatively classified into cellular and particle-dispersed morphologies. The different microstructural morphologies were able to be effectively analyzed by the persistent homology analysis. From the zero-dimensional persistent homology diagrams (PD0), the size, spacing, shape, and spatial distributions of Si particles were analyzed and quantified. PD0 analysis not only demonstrated the fact that Si particles grew or were coarsened to spherical shapes with heat treatments but also that fewer particles existed near large particles. Furthermore, the cellular to particle-dispersed microstructural morphologies could be quantified using the slope of the first principal component (PC1) (direction for the maximum data variation) in the one-dimensional persistent homology diagram (PD1). The descriptors of the spatial distribution of Si particles and microstructural morphology were strongly correlated with the mechanical properties, which could be reasonable considering the load transfer effect of the Si phase.

## Figures and Tables

**Table 1:** General microstructural features (area fraction, number density, equivalent diameter, and circularity of Si particles) of as-built and subsequently heat-treated (300 °C/2 h and 530 °C/6 h) Al–12Si alloys.

Sample	Area Fraction (%)	Number Density/μm^2^	Equivalent Diameter/μm	Circularity
As-built	11.0	33.0	0.054	0.72
300 °C/2 h	13.1	30.6	0.056	0.82
530 °C/6 h	13.5	0.2	0.680	0.86

## Data Availability

Data are contained within the article.

## Supplementary Materials

The following supporting information can be downloaded at: https://www.mdpi.com/article/10.3390/ma16227228/s1, Figure S1: Images showing the thickening behaviors during the persistent homology analysis: (a) original image and the images thickened by (b) +1 pixel, (c)+2 pixels, and (d) +3 pixels. The numbers in the pixels shown in (a) indicate the Manhattan distance from the red line.
