# Supplementary material for: Persistent Homology Analysis of the Microstructure of Laser-Powder-Bed-Fused Al–12Si Alloy
Source: Materials (Basel). 2023 Nov 18;16(22):7228. doi: 10.3390/ma16227228 (PMC10673303; doi:10.3390/ma16227228)
Supplement: Supplementary file 1 [file materials-16-07228-s001.zip › materials-2691944-supplementary.pdf]

Supplementary materials

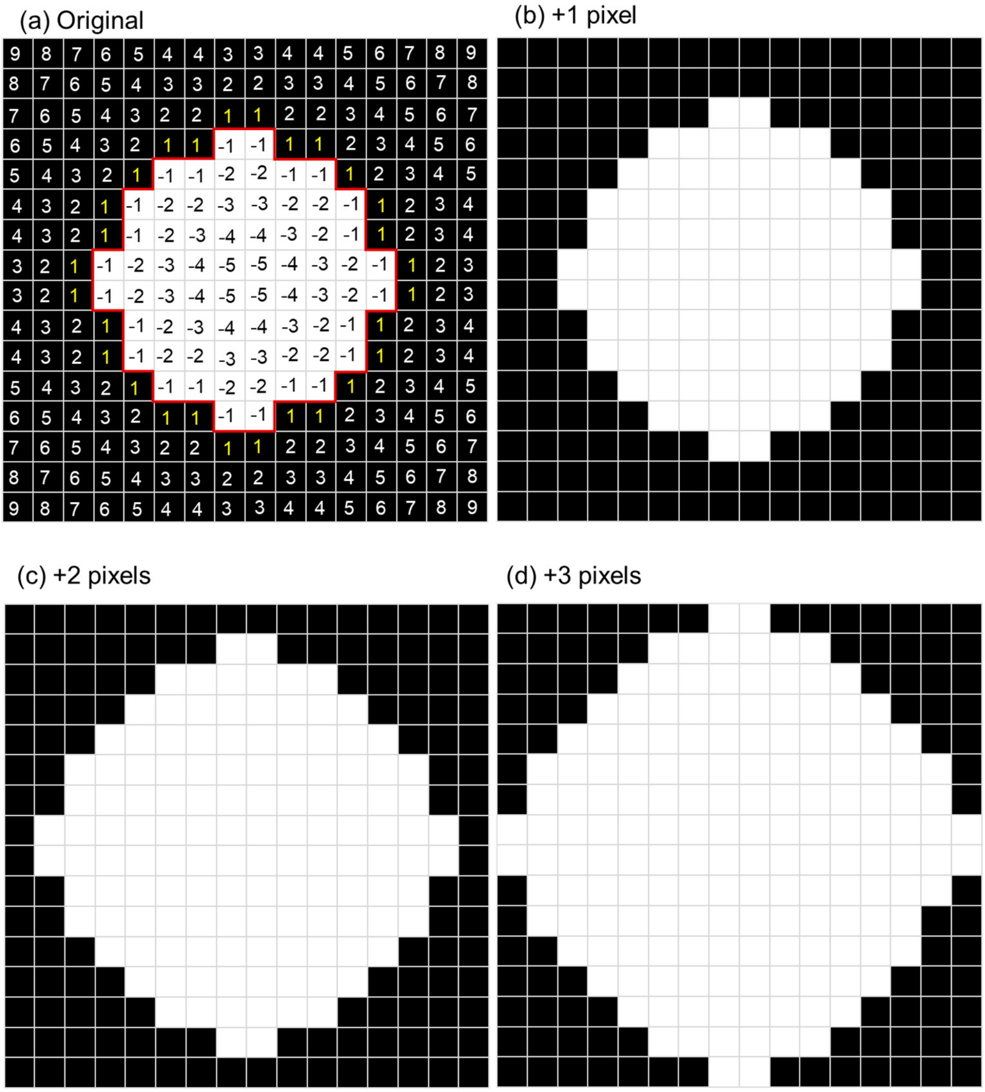

Figure S1. Images showing the thickening behaviors during the persistent homology analysis: (a) original image and the images thickened by (b) +1 pixel, (c)+2 pixels, and (d) +3 pixels. The numbers in the pixels shown in (a) indicate the Manhattan distance from the red line.
